# Barriers to medication adherence for rural patients with mental disorders in eastern China: a qualitative study

**DOI:** 10.1186/s12888-021-03144-y

**Published:** 2021-03-08

**Authors:** Xiaoyun Chai, Ying Liu, Zhuxin Mao, Shunping Li

**Affiliations:** 1 Centre for Health Management and Policy Research, School of Public Health, Cheeloo College of Medicine, Shandong University, Wenhua Xi Rd 44, Jinan, 250012 China; 2 NHC Key Laboratory of Health Economics and Policy Research, Shandong University, Jinan, 250012 China; 3 Center for Health Preference Research, Shandong University, Jinan, 250012 China; 4 Asset Management and Operation Department, Shandong Provincial Hospital Affiliated to Shandong First Medical University, Jinan, 250021 China; 5 School of Insurance, Southwestern University of Finance and Economics, Chengdu, 611130 China

**Keywords:** China, Mental disorders, Medication adherence, Qualitative research

## Abstract

**Background:**

Mental disorders are destructive and usually require long-term medication, but non-adherence of medication is highly prevalent in patients with mental disorders. Previous studies relating to medication non-adherence were mainly quantitative. Meanwhile, there have been even fewer studies conducted in rural areas in China that focused on patients’ medication non-adherence. This study aims to explore the barriers to medication adherence for rural patients with mental disorders in China from the perspectives of patients, patients’ family members and healthcare providers.

**Methods:**

A qualitative study was carried out in the rural areas of four towns within Shandong Province in eastern China. The study adheres to COREQ guidelines. Semi-structured in-depth interviews were conducted with 11 patients, 21 family members and 8 primary mental health service providers.

**Results:**

Thematic analysis generated five major themes: (1) lack of self-insight, (2) inadequate family support, (3) long treatment duration and side effects of drugs, (4) poor economic conditions, and (5) the perceived stigma of illness.

**Conclusion:**

These findings may be useful for policymakers and planners to improve medication adherence and decrease the recurrence rate of mental disorders in China.

## Background

Mental disorders include depression, bipolar disorder, schizophrenia and other psychoses, dementia, and developmental disorders including autism [1]. Some mental disorders are severe and characterized by deteriorating course of illness, which can lead to devastating consequences for young and adult patients who are at the most productive period in their lives [2]. The global prevalence of common mental disorders is 17.8% [3] and nearly one in five U.S. adults live (46.6 million) with a mental disorder [4]. The burden of mental disorders has become a major public health concern in China [5, 6], where the weighted lifetime prevalence of any mental disorder excluding dementia during the participants’ entire lifetime was 16.6% [6].

Treating persistent mental disorders is usually a long-term process, which is comprised of antipsychotics and psychosocial interventions [7]. Several studies have shown that the relapse rate is significantly lower when patients adhere to their medication regime [8, 9]. However, non-adherence is highly prevalent in patients with mental disorders. A review reported that the mean rate of medication non-adherence was 41.2% in patients with schizophrenia [10]. Another study showed that the prevalence of medication non-adherence among patients with severe mental disorder was 55.2% [11]. Non-adherence to prescribed psychoactive medication can greatly increase the risk of illness exacerbation and rehospitalization [12, 13]. It also increases the risk of attempted suicide and lowers quality of life [14].

Previous studies measuring medication non-adherence for people with mental disorders are mainly quantitative, focusing on the relationships between the occurrence of non-adherence and those influencing factors including patient-related, medication-related, illness-related, and external/environment factors [15, 16]. The information collected in those quantitative studies which used close-ended surveys can be limited, because patients’ attitudes and personal beliefs regarding mental disorders and treatment are heterogenous and not adequately captured [17, 18]. Structured, closed-end surveys do not allow patients to fully express themselves and to respond beyond the survey options [19]. There have been even fewer studies conducted in Chinese rural areas that focus on medication non-adherence for people with mental disorders. This study aims to fill the gap by exploring the barriers of medication adherence for Chinese rural patients with mental disorders using a qualitative method.

## Methods

### Study setting and participant

A series of semi-structured, open-ended interviews with patients, the family members of patients and local primary mental health service providers were conducted to attain the research aim. Consolidated criteria for reporting qualitative studies (COREQ) guided the designing and reporting of our study [20].

This study was conducted in Shandong Province, which is located in eastern China with a population of more than 100 million. We randomly selected two towns (Zhangjiawa and Xinzhuang) from one central city (Laiwu), and two towns (Nigou and Xiji) from one southern city (Zaozhuang).

Qualitative interviews were conducted with 11 patients with mental disorders, 21 family members of patients and 8 local primary mental health service providers. The inclusion criteria for patients are as follows: (I) diagnosed with one type of mental disorder, (II) age >18 years, (III) had a course of disease lasting at least 3 years, (IV) certified by the doctor to have the cognitive ability to communicate and participate in qualitative interviews. There was no refusal of consent or drop-out during interviews. Participants’ demographic information is presented in Table 1.

**Table 1:** Demographic information of participants

	Patient	Family member
Age, year (mean, SD)	46.1 (13.5)	60 (10.4)
Range (age)	28–69	35–81
Female (n, %)	4 (36.4%)	12 (57.1%)
Duration of illness, year (mean, SD)	17.4 (11.0)	–
Education (n, %)		
Uneducated	2 (18.1%)	7 (33.3%)
Primary school education	4 (36.4%)	7 (33.3%)
Junior middle school education	4 (36.4%)	5 (23.8%)
High school degree or above	1 (9.1%)	2 (9.6%)
Marital status (n, %)		
Married	6 (54.5%)	21 (100%)
Single	4 (36.4%)	–
Divorced	1 (9.1%)	–
Living condition (n, %)		
Living with others	10 (90.9%)	–
Living alone	1 (9.1%)	–
Diagnosis (n, %)		
Schizophrenia	8 (72.7%)	–
Depression	2 (18.2%)	–
Bipolar disorder	1 (9.1%)	–

### Data collection

Interviews were conducted in November 2018 for two weeks by XY C, Y L and SP L in the local dialect. All interviewers were trained to be competent to undertake qualitative interviews on sensitive topics. All participants were interviewed in healthcare institutions arranged by community partners. One-to-one interview was held in a private room, allowing participants to speak freely and comfortably. The interview process lasted 25 min on average and was audio recorded. The interview started with simple open questions, such as “How are you feeling recently?”; “Are there any difficulties or discomfort recently?”; “Does your doctor prescribe you any medicine?”, followed by in-depth discussions based on respondents’ answers. This could add flexibility to explore issues that emerged during discussions. In our interview, not all questions were open ended, this was because, on the one hand, rural patients and their families did not attain enough education, too many open questions can divert their attention, bring about additional participant burdens and finally cause difficulty for participants to complete interviews. On the other hand, although some of our questions were less open ended, we did not ask any leading questions. The study was approved by the Medical Ethics Committee of School of Health Care Management, Shandong University (ECSHCMSDU20181103).

### Data analysis

All interviews were transcribed verbatim. The transcripts were analyzed using thematic analysis [21]. XY C who is familiar with the dialect proofread recordings and transcripts, ensuring the data validity after the translation of dialect and colloquialisms. A preliminary code structure, including possible themes and specific codes, was developed based on relevant literature. The code structure was revised after XY C and SP L read all the transcripts. ZX M and Y L participated in finalizing the code structure. Based on the finalized code structure, XY C and Y L undertook coding and thematic consolidation. Any differences arising from the whole work were resolved by consensus. The numbers of interviews were designed to enable a theoretical saturation of qualitative themes, and data collection ceased at a point when no new information was obtained. The data analyses were conducted in Excel. All analyses were performed in Chinese and were translated into English for reporting.

## Results

### Key themes

Five major themes were generated: (1) Lack of self-insight; (2) Inadequate family support; (3) Long treatment duration and side effects of drugs; (4) Economic conditions discouraging them from purchasing medicines; (5) The perceived stigma of illness. Each theme is discussed below, supported by quotations from participants.

### Lack of self-insight

More than half of patients with mental disorders were admitted to hospital with an acute illness. Most of them were not aware of or simply did not understand their mental disorders. They seemed to refuse to admit that they were mentally ill, so they refused to take medicine.

*Before, we couldn’t control (him). He always threw the bowl on the ground when we tried to give him the drugs. Never admitted he was ill. Sometimes he said: “I’m not ill! Why would I be ill!! I AM NOT!! You can go to the hospital if you want. I don’t want to be hospitalized!” (Family Member 16, 65 years)*

*I hated the medicine when I was younger. Why would I need it when I am ok? (Patient 04, 28 year)*

*If a doctor gives him the medicine and watches him, he takes it. But at home, he refuses the med we give him. He thinks he is perfectly healthy. (Family Member 10, 53 years)*

On the other hand, some patients might be depressed, anxious and angry at the time of onset, it would be difficult to control their behaviors in such states, so they were less likely to take drugs on time.

*I don’t know it when I lose my consciousness. I don’t know if I should take the meds or not. I just don’t have consciousness of what I’m doing. (Patient 05, 44 years)*

*I usually take drugs on time. But can’t control myself when I have an attack. Can’t remember to take them. (Patient 11, 37 years)*

*Sometimes it’s not that she forgets things. Sometimes we want her to take the meds and she’s not stable. So she doesn’t take it. (Family Member 25, 48 years)*

### Inadequate family support

In the treatment of mental illness, the support provided by family members was often essential. Patients often forgot to take their medication if their family members did not remind them. Almost all family members who took care of patients mentioned this point.

*I saw that he (the patient) took only one drug at the hospital and took 3 drugs after back at home. But he doesn’t take it if no one is watching. (Family Member 11, 56 years)*

*(The patient) hasn’t taken the meds. His two children are not home. Who should feed him? (This patient has never taken any medication) (Family Member 28, 60 years)*

Some patients were in their youth when they were ill. They were supposed to provide financial income for their families, especially in rural China and needed to go to work. However, since the side effects of taking drugs, such as fatigue and sleepiness, would affect their normal work, their family may intervene or even stop those patients’ taking their medicine.

*At home we can’t be as attentive as when they are at the hospital. We expect him to help with the farming, so we don’t give him the meds in the morning, otherwise he might not be able to work. (Family Member 16, 65 years)*

*I find in my follow-up visits that they have something in common. When they’re at the hospital and accompanied by professionals, they take the medicine regularly. But things are bound to change when they return home. Last Friday I visited one patient at his home. He’s got mania but he’s the main labor force of the family. The family is raising many pigs. When he takes the meds, he’s very sleepy and cannot help with the work. So his family cut down on his meds and sometimes even doesn’t give him the meds. (Medical Staff at Nigou Town, 40 years)*

Family members of patients had to go to the fields to cultivate, and sometimes the patient was left alone at home. Because family members work in farming for most of the day, it is difficult for them to regularly remind patients to take their medicine, which can lead to reduced medication compliance.

*I usually put the medicine on the table before I go out (to the fields) and come back to see if she’s taken it. If not, I watch her take it. If she refuses, I make her do it. (Family Member 31, 69 years)*

### Long treatment duration and side effects of drugs

Antipsychotic drugs needed to be taken for a long time. The length of such treatment could decrease individual patients’ medication adherence. Some patients stopped the drugs they were taking and switched to other antipsychotic drugs. Some patients simply stopped the treatment without taking any other drugs because they were too tired to continue.

*The reason of stopping the medicines could be that she is sick of the whole thing, taking the medicine every day, was uncomfortable and doesn’t want the meds. (Family Member 14, 46 years)*

*It might be that the treatment has been too long. He’s got tired of it and doesn’t want to take the meds any more. (Family Member 12, 63 years)*

Many patients mentioned that they would have some uncomfortable symptoms after taking the medicine. For example, after taking drugs, patients might become sleepy and have dry mouth, blurred vision` and other symptoms, which could affect their normal life, especially when the symptoms were serious.

*Sometimes I don’t feel well after take medicine. I can’t express how uncomfortable I felt after I took the medicine, Sometimes I feel dizzy. I don’t want to take medicine. (Patient 07, 61 years)*

*There are also personal reasons (for not taking medicines). If he doesn’t feel well after taking the meds, he secretly stops it. (Mental Health Worker at Nigou Town, 40 years)*

### Economic conditions discouraging them from purchasing medicine

A few patients reported that they could only reimburse half of the cost of treatment. As a result, in the process of medical treatment, their family had to bear the remaining cost. The family was likely to owe a debt for the patient’s drugs. Since they might not afford the whole treatment process, they could be forced to accept intermittent treatment, where the continuity of drugs was not guaranteed.

*How could he afford it? He doesn’t even have basic living allowance. He’s spent all his money to treat this disease. That treatment in Beijing alone costed over 30000 yuan (about $4500). The medicines are all very expensive. The medicines bought in Beijing were over 100 yuan (about $14) each package! Couldn’t afford the expensive medicines so he bought cheap ones. Last year he applied for the basic living allowance but didn’t get it. He’s only 15 or 16 years old. Poor child! And he couldn’t even get the allowance. (Family Member 18, 57 years)*

*Some have poor economic conditions. If they don’t have basic living allowances or for some other economic reasons, they might stop the medicines. (Mental health worker at Zhangjiawa town, 50 years)*

Similarly, some patients mentioned that the diseases had caused a heavy financial burden. They had to stop taking medicine, or chose to take medicine when their condition was serious, but stop taking medicine after their condition had been alleviated.

*The treatment alone was over 8000 yuan (about $1126) and there were other fees too. We could no longer afford it so we didn’t get further treatment. (Family Member 20, 74 years)*

*I couldn’t afford it all and bought some medicines. They didn’t last long. When I were having an attack later, I couldn’t even have meds. (Patient 11, 37 years)*

### The perceived stigma of illness

Some patients regarded buying psychotropic drugs or taking drugs as a stigma. In people’s understanding of mental disorders is less comprehensive in China’s rural areas, which may result in discrimination and even insults on patients.

*He’d throw away the meds secretly. He wouldn’t take the name of a “mental” from others. He’d punch them. (Family Member 10, 53 years)*

*Some patients are resistant to their diseases and can easily get irritated if they hear people talk about them. And some family members lock the patients inside the houses when they go to the fields, even though the condition of patients is not that serious. But the family members cannot put their minds at ease if not locking the patients. They fear the patients might go out and get into trouble. And being locked in also irritates the patients. It’s not good for their recovery. (Mental Health Worker at Xiji Town, 39 years)*

Some patients were in the age of getting marriage or getting a job. Having a mental disorder was believed to have a great impact on their interpersonal interaction. Therefore, they refused to take medicine, hoping to hide their mental disorder from others.

*I’m so worried that my boyfriend might leave me because of the disease. I used to be into my looks but now I don’t even want to wash my face. I don’t want to go out. (Patient 05, 44 years)*

## Discussion

Most mental disorders are chronic disorders with multiple relapses, which require patients to continually take medicine. The non-adherence to medicine among patients with mental disorders in China, especially in Chinese rural areas, are highly prevalent, but in-depth evaluations of such phenomenon have rarely been reported. This study used qualitative tools to explore factors affecting the compliance of Chinese rural patients. Our mix of data sources across patients, their family, and healthcare providers enabled triangulation of themes and identification of varying viewpoints. The patients of our interview were the ones who directly received treatment and they could provide us with their real experience on their medication treatment, while the patients’ families, who were living with the patients, were standing on another perspective and showed us the important impact of family support on patient’s medication adherence. The perspective of the mental service providers was a more macro perspective, as they were experts and had met and treated different patients in rural areas. Generally speaking, there were much consensus among these perspectives. The triangulation across three interview groups jointly identified factors affecting the medication adherence of patients with mental disorders in rural China, including lack of self-insight, lack of family support, long treatment duration and side effects of drugs, poor economic conditions and perceived stigma.

The lack of insight may decrease patients’ willing to fulfil the treatment and these patients may choose not to follow doctors’ prescription. Poor insight, often occurs when patients with mental disorders fail to recognize the nature of their disorder and the need for treatment [22]. In some countries, for patients who considered to be with limited capacity to consent to care themselves, treatment can be mandated to them through community treatment orders (CTOs) [23]. In the Rural Areas of China, the effective treatment of serious patients should also be strengthened. For those who refuse to take medicine during the onset period, if necessary, compulsory medicine may be introduced to help control their illness.

Our results also suggest that family support will influence the medication adherence of patient. Because there is a deep-rooted family collectivism in China’s rural areas, when a person is sick, the whole family are likely to unite to take care of the patient [24]. Some studies have shown that the burden of rural families is negatively correlated with the function of community care network, family members of patients may encounter various difficulties in providing care for patients with mental disorders, and tend to have family caregiver burden [24, 25]. The family caregivers’ burden is not only a Chinese issue but a global issue. Previous studies have shown that such burden is experienced by family caregivers of patients with mental disorders [26]. The family caregiver burden negatively affects physical and mental health [27, 28], social relationships [29, 30], and financial life [27, 29, 31, 32] of caregivers. For example, the caregiving burden can cause much marital discord, especially for women whose husbands suffer from schizophrenia [33]. Another common phenomenon in Chinese rural culture is that if patients don’t go to farming, the main income of the family can’t be guaranteed, therefore, patients have to reduce or even stop taking their medicine if it interferes with their work. Those patients who have to stop taking drugs are usually the main labor force of the family.

The predominant and recommended treatment for some mental disorders involves the continued long-term use of antipsychotic medication for both symptom control and the prevention of relapse, although continued long-term antipsychotic treatment is necessary and recommended by clinicians, many individuals with mental disorders fail to adhere to treatment [34]. Wade et al. research has indicated that between 75 and 90% of individuals discharged from hospital discontinue their medication within a year or two of discharge [35], which is basically consistent with our research results. This discontinuation results in relapse, greater functional impairment and a poorer prognosis for the individual. Therefore, when a diagnosis of mental disorders is established, it is important that the prescribing clinician clearly and effectively communicate the treatment time to the patient, which can enable the patient to have sufficient psychological preparation to persist in taking the medicine for a long time. While antipsychotic drugs do improve conditions for many patients with mental disorders and are widely used, many of them have chronic adverse effects [36]. In particularly, multiple antipsychotics is often used in mental health treatment, where patients take more than one antipsychotic at a time. Under such treatment, patients are even more likely to experience side-effects, and to receive higher than recommended antipsychotic doses [37]. Combined medication increases the risk of adverse effects [38], which may affect a patient’s daily functioning, including the ability to farm. It is necessary to further study the simplification of medication regimen and the investigation focusing on the identification and reduction of side effects [37, 39].

Economic burden is another factor. The Chinese government has launched a series of movements to reinforce community-based healthcare in patients with mental illness. In 2004, “The central government subsidizes local health funds for severe mental disorders management treatment project” (686 project) was launched to provide diagnosis, follow-up, free use of essential medicines, emergency medical treatment, free emergency hospitalization and other rescue activities for poor patients with severe mental disorders, in order to improve the cure rate and quality of life of patients and can help patients to restore social function, reduce the risk behavior of patients [40, 41]. In our interview, despite a health service provider mentioned that there were patients who signed up for the “686 Project” to get free medicines from community health service centers or hospitals. However, among the patients we interviewed, a small number of patients still reported that they could not buy enough medicines due to economic reasons. There are also cases in some areas where patients are too young or too old to be included in the 686 Project or to obtain medical insurance. Further policies need to address the economic burden among those elderly and those young people who are not within the Project scheme.

Stigma in patients with mental disorders can also be an influential barrier. As previous studies indicated, people tend to be prejudiced against patients with mental disorders, and civil society often perceives those patients as dangerous and disruptive [42, 43]. Deeply held cultural and philosophical beliefs can promote stigma, which finally acts as a barrier to rehabilitation and recovery [44]. In the Chinese cultural setting, a diagnosis of mental disorders often results in a “loss of face” for the individual [45]. Losing one’s face can lead to lower self-esteem and can damage people’s social identity [44]. Additionally, compared with advanced mental health inpatient facilities and regional rehabilitation services in countries such as Japan, China’s psychiatric hospitals are mostly located in cities, while psychiatric treatment is relatively undeveloped in rural areas [46, 47]. As a result, mental patients in rural areas are more likely to live with their families in the community and mingle with the general public in rural places, while the general education level of residents in rural communities in China is not high, which can lead to patients being more susceptible to public prejudice and stigma [48]. When a patient knows their diagnosis, they begin to internalize stereotyping and discrimination. Such inferiority complex can cause depression, refusals to take medicine, or even refusals to let their families buy medicine. This shows the need to popularize the social awareness of mental disorders and appeal to the society to respect patients with mental illness.

### Strengths and limitations of this study

The qualitative methods allowed for detailed and deep responses and triangulation across providers, patients and their family members. The findings have direct relevance to policy makers considering the development of interventions to increase the rate of medication adherence in our setting. Our purposive sampling may bias our findings to those representative of patients with lower educational levels and low uptake of services. Some social acceptability bias may have influenced the results of interviews with patients and their families, which we attempted to counterbalance with mental health managers. As a qualitative study, there are limits to generalizability beyond our setting.

## Conclusions

In summary, our study details important barriers to medication adherence for patients with mental disorders, including lack of self-insight, inadequate family support, treatment duration and side effects of drugs, economic burden, and the perceived stigma of illness. These findings may be useful for policymakers and planners to improve medical services, improve medication adherence and decrease the recurrence rate of mental disorders in China. As a qualitative study, the findings documented here related primarily to our study setting, while our recommendations should be tested in further studies for wider implications.

## Data Availability

The dataset used and analyzed during the current study are available from the corresponding author on reasonable request.
